# Kidney tumoroid characterisation by spatial mass spectrometry with same-section multiplex immunofluorescence uncovers tumour microenvironment lipid signatures associated with aggressive tumour phenotypes

**DOI:** 10.1038/s44303-025-00106-x

**Published:** 2025-09-16

**Authors:** Hazem Abdullah, Greice M. Zickuhr, In Hwa Um, Alexander Laird, Peter Mullen, David J. Harrison, Alison L. Dickson

**Affiliations:** 1 School of Medicine, University of St Andrews, North Haugh St Andrews, UK; 2 Department of Urology, Western General Hospital, Edinburgh, UK; 3 NuCana plc, Edinburgh, UK

**Keywords:** Molecular imaging, Fluorescence imaging, Cancer imaging, Immunohistochemistry, Cancer imaging

## Abstract

Renal cell carcinoma (RCC) incidence is rising, and treatment remains challenging unless surgery is curative. Tumour heterogeneity contributes to resistance against both chemotherapy and immune checkpoint inhibitors, underscoring the need to better understand the complex tumour microenvironment (TME). While tumour models derived from cancer tissue from patients have advanced cancer research, they often fail to capture functional RCC heterogeneity and key TME components. We developed a 3D model system with a high success rate from resected tumour, retaining cancer, stromal, and immune cell populations. This system is fully compatible with advanced imaging technologies, including mass spectrometry imaging (MSI) and live-cell multiplex imaging. By integrating static spatial analysis with dynamic live-cell visualisation, our system provides unique insights into tumour heterogeneity, microenvironment metabolic crosstalk, and real-time cellular responses. Phenotypic characterization of the tumoroids showed strong histological resemblance to the original resected tissue, indicating that the tumoroids are reflective of the tumour in vivo and suitable as a representative model system. Additionally, DESI-MSI revealed distinct lipidomic profiles within patient-derived ccRCC tumoroids, capturing spatial metabolic heterogeneity reflective of the primary tissue. Lipid signatures varied across tumour regions, with phospholipid subclasses distinguishing epithelial, endothelial, and highly proliferative cell populations. Notably, non-clear cell regions exhibited reduced lipid droplet and fatty acid content, aligning with aggressive tumour phenotypes.

## Introduction

Kidney cancer, primarily renal cell carcinoma (RCC), accounts for ~295,000 new cases and 134,000 deaths annually worldwide^1^. Among its subtypes, clear cell RCC (ccRCC) is the most prevalent (~75%) and is distinguished by mutations in the VHL gene, which drive metabolic reprogramming and lipid accumulation, resulting in its characteristic clear cytoplasm morphology^2–4^. In addition to these metabolic changes, ccRCC is often notable for high T-cell infiltration; higher nuclear grading and stage correlate with elevated infiltration of T helper 2 and T regulatory cells^5,6^. These features have made immune checkpoint inhibitors central to the treatment of advanced and metastatic ccRCC^7,8^.

Despite significant advances in our understanding of the pathophysiology of ccRCC, clinical outcomes remain highly variable. Notably, in contrast to other cancers, high immune infiltration in ccRCC unexpectedly may correlate with poor outcomes in patients receiving ICI therapy^9^. Precision oncology aims to identify effective treatment strategies for an individual patient by studying their specific tumour characteristics, typically through genetic screening or xenograft models derived from tissue from patients^10,11^. However, tumours develop in complex and dynamic microenvironments that influence their growth, invasion, and metastasis^12,13^. Several organoid systems have been developed to mimic key aspects of organ structure and function, but they are limited. Drawbacks include the loss of heterogeneity, absence of vascular and immune components, and, crucially, restricted compatibility with mass spectrometry imaging methods, which would allow for a deeper understanding of functional metabolic resistance mechanisms^14^.

Here, we describe a novel 3D tumoroid model derived from cancer tissue from patients with ccRCC that retains the complex histological and cellular architecture of the original tumour and viable immune cells. This system enables detailed spatial and molecular characterisation using advanced imaging modalities, including mass spectrometry imaging (MSI) and live-cell imaging. By integrating desorption electrospray ionisation mass spectrometry imaging (DESI-MSI) with multiplex immunofluorescence and standard histochemical staining on the same tissue section^15^, we comprehensively characterised the histological, cancer cell and immune phenotypes, and their lipidomic environment.

## Results

### Collection and sample preparation of patient-derived tumoroids

**Fig. 1:**
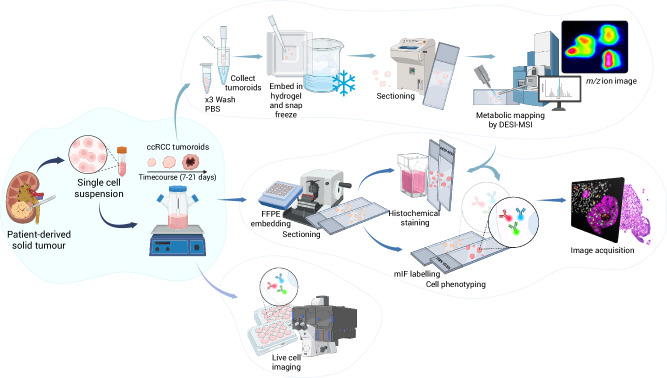
Schematic representation overview of the preparation and characterisation of RCC patient-derived tumoroids. Primary tumours were digested to form a single-cell suspension and cultured into tumoroids under optimised conditions. Tumoroids were subsequently harvested as appropriate for downstream analyses including DESI-MSI, multiplex immunofluorescence, and live cell imaging.

Multicellular tumour 3D models (tumoroids) were generated from tissue from patients undergoing resection of primary renal tumours. They were characterised histologically, phenotypically and metabolically (Fig. 1). Human epidermal growth factor (hEGF) and Y27632 ROCK inhibitor were found to be essential to induce rapid tumoroid formation, which was observed as early as 2 h post-digestion. In cases with limited tissue and therefore low cell density, initial cell clustering required up to 24 h. Long-term maintenance and expansion were successful in all cases (*n* = 8, Supplementary Table 1) for up to 21 days. (Supplementary Fig. 1). Extended culture durations were not assessed.

### Tumoroids retain histological features and heterogeneity of primary ccRCC tissue

**Fig. 2:**
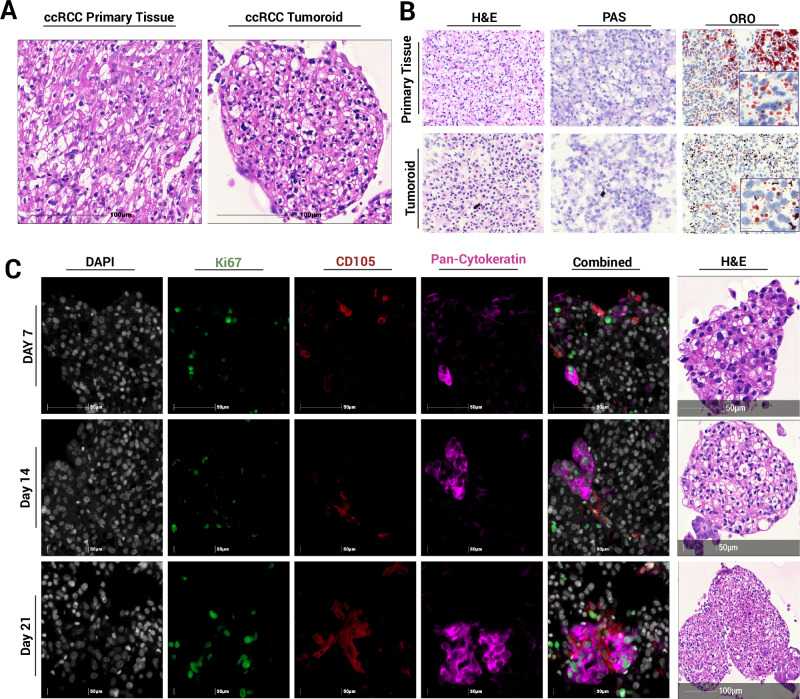
Histological and phenotypic characterisation of ccRCC patient-derived tumoroids. **A** H&E staining of FFPE primary tissue and its corresponding tumoroid. **B** H&E, PAS and ORO staining of primary tissue and tumoroids after 7 days of culture. **C** mIF time course demonstrating sustained proliferation and spatial heterogeneity of endothelial (CD105^+^) and PanCK^+^ cells.

Haematoxylin and eosin (H&E) staining of clear cell renal cell carcinoma (ccRCC) tumoroids revealed abundant clear cytoplasmic vacuoles, recapitulating the characteristic clear cell morphology of the original tumour (Fig. 2A). Histological analysis showed intra- (supplementary Fig. 2) and inter-tumoroid heterogeneity. Positive oil red O (ORO) staining of the cytoplasmic vacuoles confirms neutral lipid retention while periodic acid–Schiff (PAS) staining further indicates glycogen accumulation within these vacuoles, consistent with ccRCC pathology (Fig. 2B, Supplementary Fig. 3).

Tumoroid cells retained their proliferative capacity, with Ki67 positivity and continued growth over the three-week culture period. CD105^+^ (endoglin) cells were detected at the tumoroid periphery, and these may represent either endothelial or cancer cells^16,17^, some of which are Ki67 positive. RCC tumoroids exhibited morphological heterogeneity, consistent with the corresponding primary resected tumour. Cytokeratin 7 (CK7), typically focally or weakly expressed in clear cell RCC, was more pronounced in eosinophilic regions with high-grade nuclei^18^. Clusters of proliferative cancer cells, marked by pan-cytokeratin positivity, expanded over time (Fig. 2C).

### Preservation of a representative immune cell subtype population within tumoroids

**Fig. 3:**
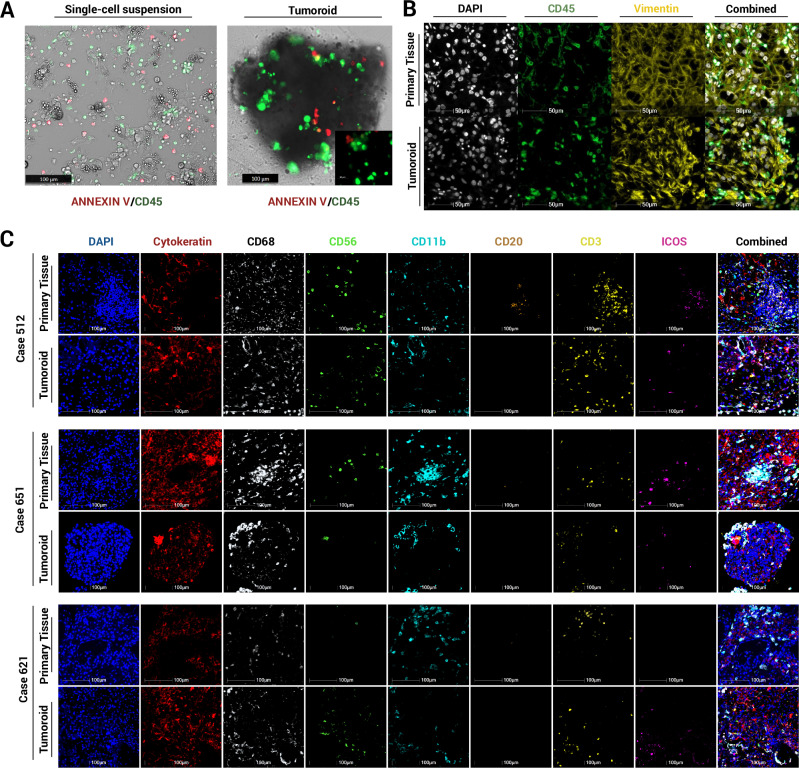
Live cell imaging and mIF panels of immune subpopulations in primary resected tumours and corresponding tumoroids. **A** Live cell imaging of single cell suspension and tumoroids showing apoptotic regions (annexin V, red) and immune cell presence (CD45, green). **B** mIF of FFPE primary tumour and tumoroids confirming immune (CD45, green) and mesenchymal, possible endothelial (vimentin, yellow) cell populations. **C** FFPE sections of primary tumours and matched tumoroids at Day 21 (*n* = 3) stained for six immune cell markers (CD68, CD56, CD11b, CD20, CD3, and ICOS) alongside the epithelial cell marker cytokeratin (CK).

Live cell imaging of tumoroids revealed diverse cell populations. Annexin V marked apoptotic cells, while CD45 identified immune cells. A single-cell suspension of collagenase-digested cells was seeded into chamber slides to assess digestion efficiency before establishing tumoroids in 3D spinner flasks. After 21 days, tumoroids were harvested and imaged using the same markers, confirming the retention of the CD45^+^ immune cell population (Fig. 3A, Supplementary Movies 1 and 2). To further verify the presence of immune cells, tumoroids were fixed, embedded in agarose and processed for FFPE. Multiplex immunofluorescence (mIF) of tumoroid sections (Fig. 3B) identified CD45^+^ leucocytes.

An 8-marker mIF panel was applied to a single section of the primary tissue and corresponding tumoroid (Fig. 3C), demonstrating that the tumoroid system preserves the immune diversity of the original tumour, maintaining a representative microenvironment. Additionally, retention of a diverse immune cell population was demonstrated through quantitative immune profiling of representative tumoroids (Fig. S4). When compared to the original tissue, CD68^+^ and CD3^+^ T cells were maintained, while CD20^+^ late B cells were absent, likely due to their terminal differentiation^19,20^. CD11b, an indicator of immune cell adhesion as well as granulocytes and macrophages^21–23^, was prominently expressed on CD68^-^ cells, suggesting the presence of granulocytes and CD56^+^ NK cells was also detected and observed within the tumoroids. Increased ICOS expression within the tumoroids indicated T cell activation^24^. These findings confirm that tumoroids preserve diverse immune subsets observed in the patient-derived tissue.

### DESI-MSI characterisation of lipidomic heterogeneity in tumoroids

**Fig. 4:**
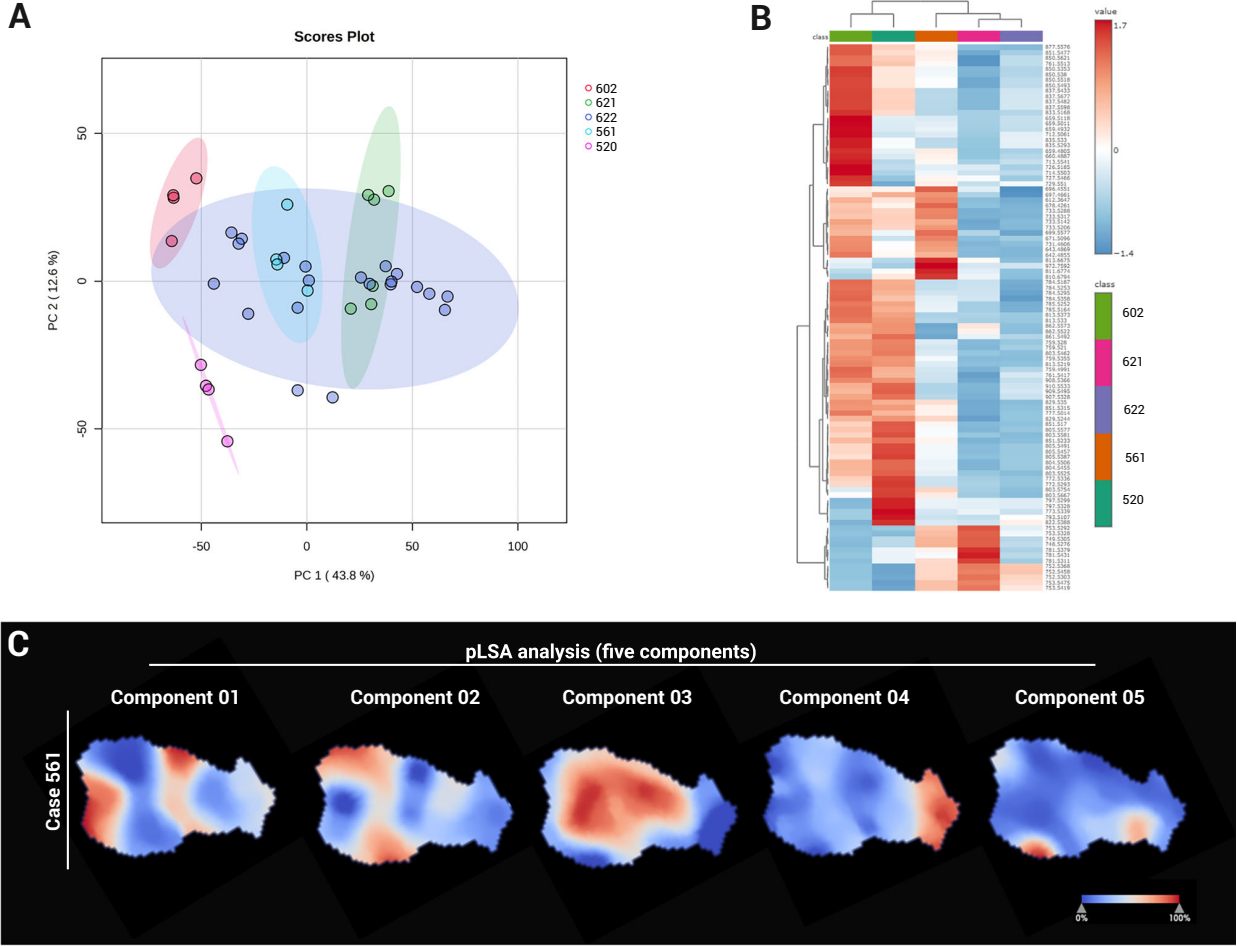
Metabolic and lipidomic heterogeneity in ccRCC tumoroids. **A** PCA scores plot of lipidomic profiles from ccRCC tumoroids derived from tumours resected from five patients. **B** Heatmap of lipidomic profiles across tumoroids (data presented as ROI average). **C** pLSA analysis of metabolites and lipids in tumoroid from case 561.

ccRCC tumours exhibit significant cellular heterogeneity, a characteristic reflected in our cultured tumoroids (Fig. 2C). To assess metabolic and lipidomic variability, tumoroids derived from patients (*n* = 5) were analysed by DESI-MSI at a 20 µm pixel resolution. Regions of interest (ROIs) comprising 260 pixels per sample were selected to capture the full area of the smallest tumoroids (~500 µm) while avoiding edges to prevent spectral artefacts. In cases where tumoroids exceeded this size, multiple ROIs of identical dimensions were selected to ensure representative sampling. This approach yielded 39 ROIs and 1090*m/z* features (*m/z* 600–1000). Principal component analysis (PCA) revealed distinct patient-specific clustering (Fig. 4A) with cases 621 and 622 forming subgroups across culture time points (days 7, 14, and 21; Supplementary Fig. 5). Ward’s hierarchical clustering analysis (HCA) further distinguished tumour cases, based on lipid profiles, identifying two main clusters: cases 602 and 520, and cases 622, 621 and 561, consistent with PCA analysis and highlight lipidomic heterogeneity (Fig. 4B, Supplementary Fig. 5).

**Fig. 5:**
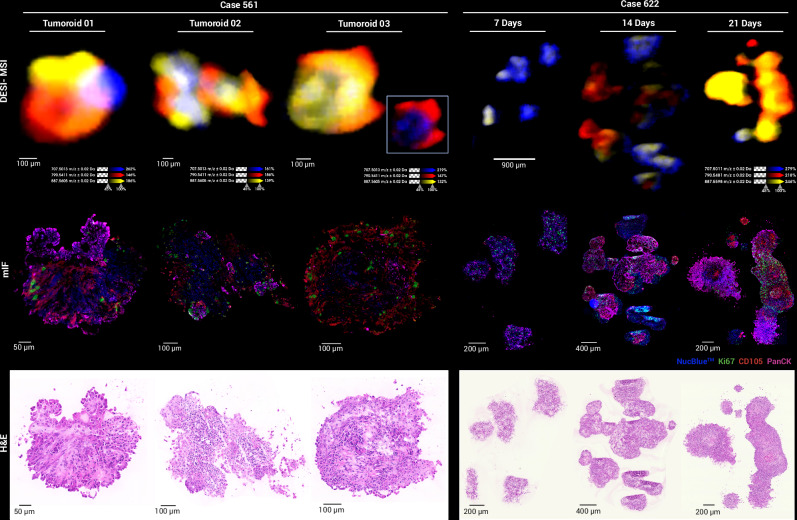
Visualisation of lipidomic heterogeneity in ccRCC tumoroids. DESI-MSI ion images of *m/z* 707.5013 (PA O-38:5, blue), *m/z* 790.5411 (PE 40:6, red) and *m/z* 887.5606 (PI 38:3, yellow) showing distinct lipid distributions in tumoroids. H&E and mIF images post-DESI-MSI reveal regions with proliferative cells (Ki67, green), endothelial cells (CD105, red) and PanCK^+^ cells (pink).

Unsupervised probabilistic latent semantic analysis (pLSA) was performed based on the spatial distribution of metabolites and lipids in each tumoroid. Detected molecular species were reduced into three to five fundamental components, highlighting inter-molecular heterogeneity (Fig. 4C, Supplementary Figs. 6 and 7). Molecular drivers contributing to spatial heterogeneity and distinguishing specific tumoroid regions were determined based on pLSA loading plots, and a receiver operator characteristic (ROC) analysis, with an area under the curve (AUC) cutoff of 0.75, highlighted molecules with similar spatial patterns. In cases 561 and 622, certain lipids co-registered to the subsequent H&E stain and were localised to delineated areas of the tumoroids (Fig. 5). To further explore molecular-phenotypic associations, we applied an mIF panel (PanCK, CD105 and Ki67) to the sections analysed by DESI-MSI, enabling co-registration of molecular distribution to phenotypical features. Importantly, all MSI, H&E and mIF data were acquired on the same tissue section, allowing for an unbiased correlation across imaging modalities. Image co-registration and alignment were performed in SCiLS Lab 2025a.

Cases 621, 622 and 561 exhibited strong PanCK and CD105 positivity, whereas cases 602 and 520 did not (Fig. 5), consistent with the observed morphological heterogeneity. Tumoroids from cases 622 and 561 demonstrated cellular diversity (Fig. 2), with some regions containing PanCK^+^ cancer cells and others lacking PanCK expression. Overlaying mIF images with DESI-MSI data, we identified molecules that spatially co-localised with regions defined by CD105^+^, PanCK^+^ and CD105^−^PanCK^−^ profiles in cases 622 and 561 (Supplementary Tables 2–4).

In case 622, tumoroids cultured over three weeks displayed an increase in PanCK and CD105 expression over time (Fig. 5). This pattern was also mirrored in lipid ion images (e.g. *m/z* 707.5011 and 790.5481, Fig. 5 and Supplementary Fig. 8), which corresponded to distinct histological regions. Notably, this case also showed a high number of proliferative cells, even at 21 days in culture. These observations prompted further characterisation of the sample, with mIF images used as a guide selection of ROI for further MSI analysis.

We compared proliferative regions based on epithelial characteristics and the presence of CD105-expressing cells, which are commonly associated with endothelial or other stromal cells. In RCC, however, CD105 has also been shown to be expressed in tumour cells and is associated with stem cell-like characteristics^16,17^. Specifically, we analysed regions that were either epithelial with CD105^+^ cells (PanCK^+^CD105^+^Ki67^High^) or non-epithelial with few to no CD105^+^ cells (PanCK^-^CD105^-^Ki67^High^). Additionally, we assessed proliferation within PanCK^+^ CD105^+^ regions by comparing areas with a high or low Ki67^+^ proliferative cell presence (PanCK^+^CD105^+^Ki67^High^ vs. PanCK^+^CD105^+^Ki67^Low^), as well as in non-epithelial, CD105 low regions (PanCK^−^CD105^−^Ki67^High^ vs. PanCK^-^CD105^-^Ki67^Low^, Supplementary Fig. 9). DESI-MSI ion selection was based on ROC area under the curve (AUC) threshold of 0.85. While PCA analysis distinguished PanCK^+^CD105^+^Ki67^High^ from PanCK^+^CD105^+^Ki67^Low^ (Supplementary Fig. 10), no features met the AUC cutoff. In contrast, low proliferative PanCK^-^CD105^−^ regions were distinguishable by 13*m/z* features (Supplementary Table 5) including three sulfatides, SHexCer 42:1;O2 (*m/z* 890.6348), SHexCer 40:1;O2 (*m/z* 862.6080) and SHexCer 42:2;O2 (*m/z* 888.6253) – which were enriched in the Ki67^Low^ areas, while phosphatidylethanolamine (PE) lipids (e.g. PE 34:0, 34:1 and 36:1) were higher in Ki67^High^ regions. A total of 47 discriminative ions were identified between PanCK^+^CD105^+^Ki67^High^ and PanCK^-^CD105^-^Ki67^High^ regions (Supplementary Table 6). PE and cardiolipins (CL) were higher in epithelial and CD105-expressing regions (PanCK^+^CD105^+^Ki67^High^), while phosphatidylglycerol (PG) and phosphatidylserine (PS) lipids were higher in non-epithelial, non-CD105-expressing regions (PanCK^-^CD105^-^Ki67^High^).

### Comparative lipidomic profiling of tumoroids and primary resected tissue

**Fig. 6:**
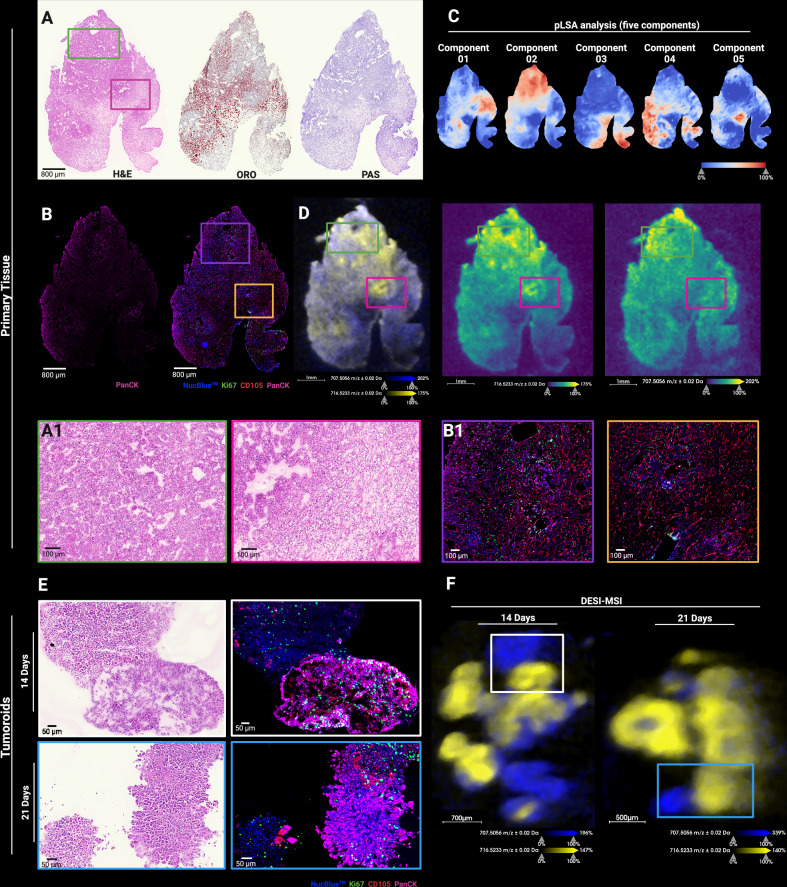
Characterisation of primary resected RCC tissue and tumoroids (case 622) using DESI-MSI, histochemical staining and mIF. **A** H&E, ORO and PAS staining of primary tissue. **A1**, **B1** Magnified regions of primary tissue of cancer cells presenting with dedifferentiated (green and purple) or high extracellular matrix-enriched (pink and yellow) patterns. **B** Primary tissue mIF images of PanCK (pink) and a composite of NucBlue™ (blue), Ki67 (green), CD105 (red) and PanCK (pink). **C** pLSA of lipids and metabolites. **D** and **F** DESI-MSI ion images of *m/z* 707.5056 [M–H]^−^ and *m/z* 716.5233 [M-H]^−^ of primary tissue and tumoroids, respectively, with highlighted regions corresponding to H&E and mIF magnifications (**A1**, **B1** and **E**). **E** H&E and mIF images of tumoroids showing cancer cells with variable PanCK expression.

DESI-MSI was performed on the primary tissue of case 622, followed by H&E staining and mIF. Histological evaluation determined cancer cell heterogeneity, with regions displaying more eosinophilic, compact cytoplasm, indicative of dedifferentiation (Fig. 6A and A1). mIF confirmed high PanCK expression with elevated levels in dedifferentiated regions (Fig. 6B and B1). pLSA analysis revealed a heterogeneous spatial distribution of metabolites and lipids (Fig. 6C). Components 02 and 04 aligned with dedifferentiated regions and elevated PanCK expression, while component 03 corresponded to areas richer in extracellular matrix and lower glycogen storage. Dedifferentiated regions also exhibited smaller neutral lipid droplets (LD) (Fig. 6A, C and Supplementary Fig. 11).

Lipidomic profiling showed that features with higher intensity in PanCK^+^CD105^+^ and PanCK^-^CD105^-^ tumoroid regions overlapped with both PanCK^+^ and PanCK^−^ areas in the primary resected tissue. Notably, lipids abundant in PanCK⁺ tumoroid regions closely matched those in dedifferentiated tumour areas (Fig. 6D, F; Supplementary Fig. 10). Both tissue and tumoroids displayed PanCK⁺ cells with variable nuclear staining intensity (Fig. 6). Fatty acid abundance, particularly long-chain fatty acids, was lower in PanCK⁺ regions, correlating with reduced LDs, while PanCK^−^ tumoroid regions retained LDs and showed higher fatty acid levels (Supplementary Fig. 11). These findings demonstrate that renal tumoroids recapitulate the lipidomic profile of their primary resected cancer from which they were derived.

## Discussion

While targeted therapies addressing common molecular alterations are now standard first-line treatments for advanced RCC, resistance often develops over time^25,26^. Both inter- and intra- tumour heterogeneity can contribute to treatment failure and resistance. To explore this heterogeneity, we developed an innovative method for culturing RCC tumoroids using a spinner flask approach. These tumoroids successfully reproduced key features of the primary tumour microenvironment, making them a valuable model system for studying RCC.

Traditional passaged organoid cultures, primarily composed only of epithelial cells, fail to mimic the complex tumour microenvironment, lacking key components such as vascular endothelium, other stromal and immune cells^27^. Recent advancements have added immune cells to organoids, for example, human intestinal immuno-organoids (IIOs), which integrate tissue-resident memory T (TRM) cells into the epithelium^28^. These models focus on a defined immune cell subtype, whereas our ccRCC tumoroids retain multiple immune subtypes of the tumour microenvironment (Fig. 3). Immune checkpoint inhibitors (ICIs) are now standard treatment for RCC and have improved patient outcomes by stimulating the immune system to target cancer cells. Our RCC tumoroids offer a promising platform to study these therapies^29^, compared to reports of organoids that lack other components of the TME^30,31^. Future studies are planned to explore functional immune modulation within the system, including ex vivo treatment of tumoroids with immune checkpoint inhibitors (e.g., anti-PD-1 or anti-CTLA-4 antibodies), with response readouts via live-cell imaging, spatial transcriptomics, DESI-MS imaging and multiplex immunofluorescence. This will allow assessment of T cell activation, proliferation, and apoptosis in response to ICIs, enabling the model to function as a testbed for immunotherapy response prediction.

Altered metabolism is a major hallmark of kidney cancer, driving tumour initiation, proliferation, and progression^32,33^. Lipid regulation plays a major role, supporting energy production, membrane integrity and signalling pathways that promote growth and migration^34^. Using DESI-MSI alongside mIF and H&E, we characterised the ccRCC patient-derived tumoroids, identifying lipid signatures associated with distinct cancer cell populations within the tumour microenvironment.

Tumoroids 621, 622 and 561 exhibited a greater presence of epithelial-like, aggressive cancer cells and a higher abundance of endoglin-positive cells, resulting in distinct lipidomic profiles compared to cases 602 and 520. Lipid analysis revealed that high CD105^+^ cell presence was associated with enrichment in glycerophosphoglycerol (PG) and highly unsaturated (≥5 double bonds) or plasmalogen glycerophosphoethanolamine (PE) lipids, while epithelial (PanCK^+^) regions showed increased levels of PEs with shorter, less unsaturated fatty acid chains. Additionally, phosphatidylinositol (PI) lipids also varied, with PI-ether lipids with 32 and 34 FA carbon chains enriched in CD105^+^ regions, and PI 38:3 specific to PanCK^+^ areas. CD105^-^PanCK^-^ regions contained higher levels of ether-linked lipids and PI 38:4, and proliferative regions showed higher saturated and monounsaturated PE levels.

Lipid metabolic reprogramming in cancer enhances lipid uptake and accumulation, contributing to tumour growth and immune evasion^35^. PE, the second most abundant phospholipid in mammalian cell membranes, plays key roles in autophagy and mitochondrial function^36^. While global lipidomic studies indicate lower PE levels in ccRCC compared to normal kidney tissue, high-grade tumours exhibit increased PE content, correlating with reduced apoptosis and altered tumour metabolism^37^. Our findings suggest that elevated saturated and monounsaturated PE levels in PanCK^+^ and Ki67^High^ regions may be associated with higher tumour grade. Additionally, the spatial distribution of cardiolipins, mitochondria-specific lipids, in PanCK^+^ areas suggests a potential link between mitochondrial function and tumour aggressiveness, warranting further investigation.

CD105 expression in RCC is not limited to endothelial cells but is also found in tumour-associated vasculature and tumour cells^16,17,38^. RCC-CD105-expressing tumour cells exhibit stem-cell-like properties and contribute to a malignant phenotype. Cancer stem cells possess a high capacity for differentiation, self-renewal and expression of antiapoptotic mechanisms supporting migration and post-treatment relapse^16^. The higher presence of plasmalogens in areas of the tumour highly expressing CD105 in our tumoroids suggests these molecules may function as antioxidants, protecting other phospholipids, such as the identified PUFA-PEs, from oxidative stress and promoting cancer cell survival^39,40^.

A recent multi-omic profiling study identified a new subtype of ccRCC, termed de-clear cell differentiation (DCCD-ccRCC), characterised by a non-clear cell phenotype, reduced lipid droplets (LDs), and lower fatty acid (FA) levels—features linked to higher proliferation rates and poor outcomes, even in stage I tumours^41^. In our study, particularly in case 622, we observed similar non-clear cell regions within tumoroids, preserved from the primary tumour, suggesting these areas could be more aggressive and potential sites of micrometastasis. Consistent with the DCCD-ccRCC findings, these regions exhibited reduced LD and FAs (Supplementary Fig. 11). While glycogen storage, another hallmark of ccRCC, was not assessed in the DCCD cohort, it appeared preserved in these regions in our study. Additionally, these PanCK-enriched areas displayed a distinct phospholipid signature across multiple tumoroids, offering new insights into lipidomic heterogeneity in ccRCC.

By integrating multimodal imaging, we developed a reproducible 3D system that captures the cellular heterogeneity of the tumour microenvironment (TME) from patient-derived tissue. Spatial metabolic and phenotypic profiling revealed inter- and intra-tumoroid heterogeneity, distinguishing lipidomic signatures in proliferative, epithelial, and endothelial cell populations. Although metabolic profiling was performed on a relatively small patient cohort (*n* = 5), multiple tumoroids and ROIs were analysed per patient, providing biological replicates that enhanced the robustness of the characterisation. Importantly, the primary focus of the characterisation was on the co-registration of mass features within spatially defined ROIs, enabling molecular and phenotypical profiling within the TME and highlighting intra-tumoral heterogeneity. While the current sample size limits inter-patient comparisons, future studies including larger patient cohorts will be essential to validate and extend these observations.

Given that ccRCC is recognised as a disease with pronounced metabolic changes, this system provides a valuable platform for investigating therapeutic strategies. While direct correlations with clinical outcomes were beyond the scope of this study, the spatial features identified, such as in PanCK^+^ niches, may represent aggressive or treatment-resistant tumour subpopulations. Future studies involving drug-treated tumoroids will be important to assess therapy response and potential predictors of treatment efficacy. Moreover, spatial mapping of metabolites within tumoroids may improve understanding of drug responses across different niches in the TME, paving the way for more precise, personalised treatment approaches.

## Methods

### Tissue preparation and tumoroid culture

Tissue from renal cell carcinoma (ccRCC) was obtained from complete nephrectomy procedures undertaken within the Western General Hospital, Edinburgh. Ethical approval was granted by Lothian Biorepository (SR1787 10/ES/0061). Resected tissue was immediately placed in a Transfer Medium consisting of Advanced Dulbecco’s Modified Eagle Medium/F12 (Gibco #A5256701) supplemented with HEPES (Fisher #15630-080), Glutamax™ (Fisher #35050038), a penicillin/streptomycin/amphotericin B solution (Sigma A5955) and stored at 4 °C until processing (12–24 h). Tissue was washed in sterile PBS, and a representative portion was fixed in 10% formalin, and the remaining (~1 g) was diced using a scalpel and incubated in the Transfer Media further supplemented with collagenase type II (50 mg/10 mL; Gibco #17101015) and ROCK inhibitor (10 µL/10 mL; Tocris). The mixture was shaken at 200 rpm at 37 °C for 30–60 min, and a single-cell suspension was then obtained by passing the digested tissue through a 70 µm cell strainer, followed by centrifugation at 400×*g* for 5 min at 4 °C. Red blood cells (RBCs) were lysed using an RBC lysis buffer (Fisher Scientific #12770000). After further centrifugation, the remaining cells were washed three times in transfer media (400×*g*, 4 °C, 5 min each). Finally, a cell count was performed.

Cells (~10,000,000) were seeded into 100 mL spinner flasks containing 45 mL Transfer Media, 5 mL FBS (10%), 50 µL ROCK inhibitor, and 50 µL human epidermal growth factor (hEGF) (Gibco; #PHG0315). Spinner flasks were incubated at 37 °C and 5% CO_2_ for 7 days, stirring continuously at 27.5 rpm. On day 7, 50 mL of fresh Transfer Media, without ROCK inhibitor, was added. The media was partially refreshed again on day 14, with 50 mL of the culture media removed and replaced with 50 mL of ROCK inhibitor-free media. Tumoroids were cultured up to 21 days and harvested on days 7, 14 and 21 before new media was added.

### Tissue processing, embedding and sectioning

For histology and mIF, tissue was fixed in 10% formalin overnight at room temperature (RT). Tumoroids were washed in PBS and fixed in 4% paraformaldehyde (PFA) for 30 min at RT, washed in PBS, and embedded in 2% melted low-temperature agarose (100–200 µL). The tumoroids were gently mixed to ensure even distribution and then placed on ice to allow the agarose to set. Once solidified, 70% ethanol (~5 mL) was added and mixed by vortex. The embedded tissue and tumoroids were processed overnight and embedded in paraffin (Leica ASP300). FFPE tumoroids were sectioned at 3 µm thickness for H&E staining, IHC and multiplex IF.

For DESI-MSI analysis, tumoroids were washed three times in PBS. Both tissue and tumoroids were embedded in Polyvinylpyrrolidone, MW 360 (PVP) (2.5%) and modified (hydroxypropyl) methyl cellulose (HPMC) (7.5%, 40–60 cP). Sectioning was performed on a dedicated (MSI use only) HM525 NX Cryostat (Epredia, Portsmouth, USA) to a thickness of 10 µm. Serial sections were thaw-mounted onto Superfrost® microscope slides (Thermo Scientific), nitrogen-dried, vacuum-packed and stored at −80 °C. Prior to analysis, sections were equilibrated to room temperature under vacuum for 20 min.

### Mass spectrometry imaging experiments

Analysis was performed on a Xevo G2-XS Q-ToF equipped with a DESI-XS ion source and heated transfer line (Waters, Milford, USA) operated at 20,000 resolving power in negative ionisation mode between *m/z* 50–1200. A solvent mixture of 98% methanol (analytical grade, Sigma-Aldrich) and 2% water was delivered at 2 µL/min and nebulised with nitrogen at a backpressure of 1 bar. Spatial resolution was set at 20 × 20 µm. Transfer line temperature was set at 450 °C, source temperature at 150 °C, capillary voltage at 0.7 kV and scanning rate at 10 scans/s.

Data processing and visualisation were performed in HDI® (Waters, Milford, USA) and SCiLS Lab 2025a (Bruker Daltonics, Germany) and normalised to TIC. Peak picking was performed with an *m/z* window of 0.02 Da, and tentative compound assignments were made with high mass accuracy measurements (≤10 ppm mass error) using Lipid Maps®. Selected lipid identities were confirmed by on-tissue tandem mass spectrometry. Probabilistic latent semantic analysis (pLSA) was performed in SCiLS Lab 2025a, heatmap and principal component analysis (PCA) were performed in MetaboAnalyst^42^.

### H&E staining

Snap-frozen and DESI-scanned samples were treated with 10% Tween® 20 detergent (TBST) to remove hydrogel, stained with haematoxylin and eosin (H&E), and washed sequentially in water, ethanol (50%, 80%, and 100%), and xylene before being fixed with DPX (Cell Path, #SEA-1304-00A) and coverslipped. For FFPE sections, paraffin was removed via xylene and ethanol washes, followed by TBST treatment, H&E staining, and similar washes. Brightfield images were acquired after drying.

### PAS staining

Snap-frozen tissue and tumoroid sections were kept in 10% Tween® 20 detergent (TBST) for 5 min to remove hydrogel. This was followed by 1% periodic acid for 5 min and then washed in tap and distilled water prior to being covered with Schiff’s reagent (1:4) for 10 min. Sections were rinsed with warm running water for 5 min and counterstained with haematoxylin for 3 min as above. Brightfield images were acquired after the samples were dried.

### Oil red O staining

Frozen sections were rinsed in TBST wash buffer to remove the hydrogel, treated with 60% isopropanol for 5 min and stained with dilute Oil Red-O solution (3:2 in distilled water) for 12 min. Sections were rinsed in 60% isopropanol for 5 min, followed by TBST wash buffer for 1 min, counterstained with Mayer’s haematoxylin (Leica biosystems, #DS9800) and mounted on glycerol. Brightfield images were acquired immediately after mounting.

### Multiplex immunofluorescence (mIF) post DESI-MSI

H&E-stained sections were dewaxed in xylene, rehydrated and prepared in TBST prior to automated mIF labelling on a Leica Bond RX autostainer. Epitope retrieval was performed with ER2 buffer (Leica, #AR9640) for 40 min at 100 °C. Endogenous peroxidase activity and non-specific background stain were blocked by peroxide block (Leica, #DS9800) and casein blocking buffer (Sigma, #B6429), respectively. Ki67 (Agilent, #M724001-2, 1:200), CD45 (Abcam, #ab40763, 1:500) and CD105 (Human Protein Atlas, #HPA067440, 1:600) were each incubated, followed by HRP conjugated secondary antibodies (Leica biosystems, #DS9800) and were visualised using TSA fluorescein (Akoya Bioscience, #NEL741001KT, 1:200), TSA Cyanine 3 (Akoya Bioscience, #NEL744001KT, 1:200), and TSA Cyanine 5 (Akoya Bioscience, #NEL745001KT, 1:200), respectively. Cytokeratin (Agilent, #M351501-2, 1:100) was incubated, followed by biotinylated secondary antibody (Jackson ImmunoResearch, #200-002-211, 1:100) and was visualised by streptavidin Alexa Fluor 750 (Fisher Scientific, #S21384, 1:100). All antibodies are summarised in Supplementary Information Table 7. Between cycles, ER1 buffer (Leica, #AR9961, 20 min, 95 °C) was used to strip non-covalently bonded redundant antibodies. Sections were counterstained and mounted with ProLong™ glass antifade mount with NucBlue (ThermoFisher, P36985). Fluorescence images were acquired using a Zeiss Axio Scan Z1 scanner. A uniform scanning profile was used for each fluorescence channel. QuPath was used to visualise and export high-resolution images^43^.

### mIF on FFPE sections

FFPE tissue and tumoroid samples were dewaxed and dehydrated in xylene and subjected to heat-induced epitope retrieval in 0.1 M sodium citrate buffer under pressure. Endogenous peroxidase activity and non-specific binding sites were blocked by 3% hydrogen peroxide and serum-free casein. Three mIF Panels were used on the FFPE sections. For Panel 1, Primary antibodies CD3 (Agilent, #A045201-2, 1:50) CD20 (Agilent, #M075501-2, 1:50), pan Cytokeratin (Agilent, #Z0622, 1:150), CD68 (Abcam, #ab213363, 1:3000), CD56 (Cell signalling, #3576, 1:500) and CD11b (Abcam, #ab52478, 1:1000), ICOS (Abcam, #ab224644, 1:250) were sequentially incubated. Each primary antibody was followed by the appropriate secondary antibody (Alexa Fluor 488, Alexa Fluor 555, or HRP-conjugated) and visualised by TSA FITC, Cy3 and Cy5 for HRP-conjugated secondary antibodies. ICOS staining was visualised with DAB chromogen and haematoxylin (Leica Biosystems, # DS9800) and mounted using DPX (Cell Path, #SEA-1304-00A). For Panel 2, CD45 (Abcam, ab40763, 1:500) and Vimentin (Cell Signalling, 5741, 1:400) were sequentially incubated, followed by their corresponding HRP-conjugated secondary antibody and visualised using TSA FITC and Cy3. For Panel 3, Ki67 (Agilent, M724001-2, 1:200), CD105 (Human protein Atlas, HPA067440, 1:600), and Pan-Cytokeratin (Agilent, M351501-2, 1:100) were incubated sequentially, each followed by the corresponding HRP-conjugated secondary antibody and visualised with TSA FITC, Cy5, and Alexa Fluor 750. Hoechst 33342 was used for nuclear counterstaining, and ProLong™ Gold anti-fade mounting medium was applied for all three panels. Heat-induced stripping in 0.1 M sodium citrate buffer and proprietary reagents were used to remove antibodies between cycles. Digitised images were acquired as above for mIF post-DESI.

### AI-enabled image fusion and image analysis

Image fusion, brightfield and fluorescence image analysis were performed using HALO® AI v3.6.4134 (Indica Labs). Three fluorescent images (CD3/CD20/DAPI, CK/DAPI, CD68/CD56/CD11b/DAPI) and one deconvolved brightfield image (ICOS/haematoxylin) were fused to generate one high-plex IF image. Fluorescence whole-slide images were processed using the HALO® image analysis software in conjunction with HALO AI™ (Indica Labs, (https://www.indicalab.com/halo-ai). To enhance detection accuracy, a custom nuclear segmentation model was trained using HALO AI™, with DAPI staining serving as the reference for identifying nuclei. Manual annotations of nuclear and background regions were carried out on multiple samples to refine the classifier, with segmentation performance confirmed by visual inspection to reduce false detection.

Immune cell populations within these regions were analysed using the HighPlex FL™ module. Marker-specific fluorescence thresholds were applied to detect CD68, CD56, CD11b, CD20, CD3, and ICOS expression. Threshold values were standardised across samples using positive control tissues to ensure consistency in detection. The proportion of marker-positive cells was quantified for each tumoroid and visualised as stacked bar plots to illustrate differences in immune cell composition between samples.

### Live cell imaging

Single cell suspensions were seeded into 18-well chamber slides (ibidi; #81816), and 21-day-old tumoroids were manually transferred to ultra-low attachment plates (Revvity; #6055330). CD45 positive cells were detected using Incucyte Fabfluor-488 dye (Sartorius, 4745) and 0.5 µg/mL CD45 antibody (Biolegend, 304002), conjugated in the dark for 15 min with 0.5 mM Opti-Green Background suppressor. Annexin V 647 conjugate (Biotium, 29003R-5 µg) was included (0.25 µg/mL) to visualise cell death and toxicity. Tumoroids were imaged over time using Zeiss Axio Observer 7 at 37 °C and 5% CO_2_ with brightfield, Alexa Fluor 488 and Alexa Fluor 647 channels. Movies were exported using Zeiss Zen 3.0 software.

## Supplementary information

Supplementary Information_Revision2_Clean.

Supplementary Movie 1.

Supplementary Movie 2.

## Data Availability

Raw DESI-MSI files and mIF, histology images from this study have been deposited in the EMBL-EBI BioImage Archive data repository with the primary accession code S-BIAD1661 and DOI: 10.6019/S-BIAD1661.
