# Supplementary material for: Kidney tumoroid characterisation by spatial mass spectrometry with same-section multiplex immunofluorescence uncovers tumour microenvironment lipid signatures associated with aggressive tumour phenotypes
Source: Npj Imaging. 2025 Sep 16;3:43. doi: 10.1038/s44303-025-00106-x (PMC12441136; doi:10.1038/s44303-025-00106-x)
Supplement: Supplementary file 1 — Supplementary Information_Revision2_Clean. [file 44303_2025_106_MOESM1_ESM.docx]

**Supplementary Information**

**Kidney Tumoroid Characterisation by Spatial Mass Spectrometry with Same-Section Multiplex Immunofluorescence Uncovers Tumour Microenvironment Lipid Signatures Associated with Aggressive Tumour Phenotypes**

**Supplementary Table 1 Kidney cancer cases used in generation of patient-derived tumoroids.** Abbreviations: ISUP - International Society of Urological Pathology, ccRCC - clear cell renal cell carcinoma, pRCC - papillary renal cell carcinoma and ChRCC - chromophobe renal cell carcinoma, NA – not applicable.

| **Case ID** | **Sex** | **Age** | **Histology** | **ISUP Grade** | **T Stage** | **Leibovich** |
| --- | --- | --- | --- | --- | --- | --- |
| SR1040-512 | Male | 59 | ccRCC | G4 | pT3b | 8 |
| SR1040-519 | Female | 78 | pRCC | G2 | pT1b | 2 |
| SR1040-520 | Female | 79 | ccRCC | G4 | pT3a | 9 |
| SR1040-561 | Female | 74 | ccRCC | G3 | pT3a | 5 |
| SR1040-602 | Male | 76 | ccRCC | G3 | pT3b | 6 |
| SR1040-610 | Male | 73 | ChRCC | NA | pT3a | NA |
| SR1040-621 | Female | 59 | ccRCC | G2 | pT3a | 5 |
| SR1040-622 | Female | 79 | ccRCC | G2 | pT3a | 4 |

**Supplementary Movies**

**Supplementary Movie 1. Live cell imaging of a single-cell suspension from primary ccRCC tissue post-digestion.** Cells are labelled with Annexin V (red) and CD45 (green).

**Supplementary Movie 2. Live cell imaging during early-stage tumoroid formation.** Cells are labelled with Annexin V (red) and CD45 (green).

**Supplementary Figures**

**Supplementary Figure 1. Examples of successful tumoroid formation.** H&E images of FFPE sections of patient tissue and tumoroids.

**Supplementary Figure 2. H&E images of multiple tumoroids from the same parent tissue.** Histological analysis of n=1 FFPE section with intra-heterogeneity of tumoroids derived from the same patient.

**Supplementary Figure 3. H&E, PAS and ORO Histochemical staining of case 622 tumoroids harvested at days 7, 14 and 21.** Neutral lipid and glycogen accumulation in patient-derived ccRCC tumour tissue and cultured tumoroids.

**Supplementary Figure 4. Immune profiling of patient-derived RCC tumoroids by multiplex immunofluorescence.** Top panel: Representative tumoroids from three patients (512, 651, 621) were stained for cytokeratin and six immune markers (CD68, CD56, CD11b, CD20, CD3, ICOS). Scale bars = 100 µm. Bottom graphs: Quantification of immune cell populations including CD68⁺ macrophages, CD11b⁺ myeloid cells, and variable levels of CD3⁺ and ICOS⁺ T cells. CD20⁺ B cells

**Supplementary Figure 5. Metabolic and lipidomic heterogeneity in ccRCC patient-derived tumoroids.** (A) PCA scores plot of lipidomic analysis of ccRCC tumoroids derived from five patients, including culture time in cases 621 and 622. (B) Heatmap of lipidomic heterogeneity among tumoroid cases (data presented individually for each ROI).

**Supplementary Figure 6. Metabolite and lipid-driven pLSA analysis of tumoroids**. pLSA of cases 561, 621, 520 and 602 and their corresponding immunofluorescence (mIF) images showing cells positive for Ki67 (green marker), CD105 (red marker) and PanCK (pink marker). NucBlue™ - nuclear marker.

**Supplementary Figure 7. Metabolite and lipid-driven pLSA analysis of tumoroids from case 622**. Tumoroids collected at 7, 14 and 21 days of culture and their corresponding immunofluorescence (mIF) images showing cells positive for Ki67 (green marker), CD105 (red marker) and PanCK (pink marker). NucBlue™ - nuclear marker.

**Supplementary Figure 8. Expression levels of representative lipids in tumoroids of case 622 cultured over a period of 7 to 21 days.** m/z 707.5011 [M-H]^-^ (PA O-38:5), m/z 790.5429 [M-H]^-^ (PE 40:6) and m/z 887.5597 [M-H]^-^ (PI 38:3).

**Supplementary Figure 9.** **Representative regions of interest selected for guiding metabolomics data analysis** based on the expression of protein markers CD105 (endothelial cells, red marker), Pan-cytokeratin (PanCK, pink marker) and Ki67 (proliferation, green marker). Data corresponds to case 622. mIF performed on the same section post-DESI-MSI analysis.

**Supplementary Figure 10. Lipidomic differences between selected regions of interest based on the expression of protein markers CD105, Pan-cytokeratin (PanCK, epithelial cells) and Ki67 (proliferation) in case 622.** (A-C) PCA scores plot of lipidomic analysis of ccRCC tumoroids visualising differences of lipid profile based on the expression of PanK, CD105 and Ki67. (D, F) Example AUC-ROC plot for m/z 888.6253 ([M-H]^-^ SHexCer 42:2;O2 or [M+Cl]^-^ PE 44:3) and 716.5233 ([M-H]^-^ PE 34:1). (E) Ion intensity box plot of m/z 888.6253 and (H) ion image. (G) Ion intensity box plot of m/z 716.5233 and (I) ion image.

**Supplementary Figure 11. DESI-MSI and mIF analysis of primary tissue and derived tumoroids.** (A) DESI-MS ion images of lipids identified in regions of di-differentiation and higher PanCK expression in case 622 patient tissue and its respective derived tumoroids. mIF images of (B) patient tissue and (C) tumoroids. CD105 (endothelial cells, red marker), Pan-cytokeratin (PanCK, pink) and Ki67 (proliferation, green).

**Supplementary Figure 12. ORO Histochemical analysis and Fatty acid analysis of primary tissue and tumoroids at 14 and 21 days.** Fatty acid abundance, particularly long-chain fatty acids (m/z 303.2322 [M-H]^-^ (FA 20:4) and m/z 331.2631 [M-H]^-^ (FA 22:4)), correlating with reduced LDs.

**Supplementary Table 2.** Molecules identified in CD105^+^ regions in tumoroids of case 561 and 622

| ***m/z*** | **Matched *m/z*** | **ppm error** | **Ion** | **Molecule** | **Class** | **Case 561** | **Case 622** |
| --- | --- | --- | --- | --- | --- | --- | --- |
| 124.007 | 124.0074 | -3.226 | [M-H]- | Taurine | Small metabolites | Yes | No |
| 218.1027 | 218.1034 | -3.209 | [M-H]- | Pantothenic acid | Small metabolites | Yes | In 14 days |
| 237.0912 | 237.0915 | -1.265 | [M-H]- | 15(R)-hydroperoxy-EPE (HEPES) | - | Yes | - |
| 722.5125 | 722.5130 | -0.692 | [M-H]- | PE P-(16:0/20:4)* | PE | Yes | Yes |
| 746.5154 | 746.513 | 3.215 | [M-H]- | PE O-38:7 | PE | Yes | Yes |
| 773.5337 | 773.5338 | -0.129 | [M-H]- | PG 36:2 | PG | Yes | Yes |
| 774.5392 | 774.5443 | -6.585 | [M-H]- | PE O-40:7 | PE | Yes | Yes |
| 775.5445 | 775.5495 | -6.447 | [M-H]- | PG 36:1 | PG | Yes | Yes |
| 776.5518 | 776.5447 | 9.143 | [M-H]- | PS 35:0 | PS | Yes | Yes |
| 790.5411 | 790.5392 | 2.403 | [M-H]- | PE 40:6 | PE | Yes | Yes |
| 792.5502 | 792.5549 | -5.930 | [M-H]- | PE 40:5 | PE | Yes | Yes |
| 795.5414 | 795.5393 | 2.640 | [M-H]- | PI O-32:0 | PI | Yes | Not seen |
| 796.5258 | 796.5265 | -0.879 | [M+Cl]- | PS O-35:1 | PS | Yes | No |
| 817.5074 | 817.5025 | 5.994 | [M-H]- | PG 40:8 | PG | Yes | No |
| 817.5214 | 817.5236 | -2.691 | [M-H]- | PI O-34:3 | PI | Yes | Not seen |
| 819.5184 | 819.5182 | 0.244 | [M-H]- | PG 40:7 | PG | Yes | No |
| 820.5269 | 820.5265 | 0.487 | [M+Cl]- | PS O-37:3 | PS | Yes | No |
| 838.5609 | 838.5604 | 0.596 | [M-H]- | PS 40:4 | PS | Yes | Yes |
| 841.5049 | 841.5025 | 2.852 | [M-H]- | PG 42:10 | PG | Yes | No |
| 842.512 | 842.5108 | 1.424 | [M+Cl]- | PE 40:6;O | PE | Yes | No |
| 843.5191 | 843.5182 | 1.067 | [M-H]- | PG 42:9 | PG | Yes | No |
| 845.5334 | 845.5338 | -0.473 | [M-H]- | PG 42:8 | PG | Yes | No |
| 865.5043 | 865.5025 | 2.080 | [M-H]- | PG 44:12 | PG | Yes | No |
| 867.5181 | 867.516 | 2.421 | [M+Cl]- | PI O-35:3 | PI | Yes | No |

*Confirmed by MS/MS experiment

**Supplementary Table 3.** Molecules identified in PanCK^+^ regions in tumoroids of case 561 and 622.

| ***m/z*** | **Matched *m/z*** | **ppm error** | **Ion** | **Molecule** | **Class** | **Case 561** | **Case 622** |
| --- | --- | --- | --- | --- | --- | --- | --- |
| 700.5273 | 700.529 | -1.998 | [M-H]- | PE O-34:2 | PE | Yes | Yes |
| 716.5239 | 716.524 | 0.419 | [M-H]- | PE 34:1 | PE | Yes | Yes |
| 722.5125 | 722.5130 | -0.692 | [M-H]- | PE P-(16:0/20:4)* | PE | Yes | Yes |
| 742.5388 | 742.539 | -0.539 | [M-H]- | PE 36:2* | PE | Yes | Yes |
| 743.5438 | 743.541 | 3.228 | [M-2H]2- | CL 74:2 | CL | Yes | Yes |
| 744.5539 | 744.555 | -1.343 | [M-H]- | PE 36:1 | PE | Yes | Yes |
| 745.5583 | 745.557 | 1.610 | [M-2H]2- | CL 74:0 | CL | Yes | Yes |
| 764.525 | 764.524 | 1.831 | [M-H]- | PE 38:5 | PE | Yes | Yes |
| 766.5383 | 766.539 | -1.174 | [M-H]- | PE 38:4 | PE | Yes | Yes |
| 767.541 | 767.541 | -0.521 | [M-2H]2- | CL 78:6 | CL | Yes | Yes |
| 768.5461 | 768.549 | -4.164 | [M-2H]2- | CL 78:5 | CL | Yes | Yes |
| 788.542 | 788.545 | -3.424 | [M-H]- | PS 36:1 | PS | Yes | Yes |
| 789.5445 | 789.544 | 0.633 | [M-H]- | PA 43:6 | PA | Yes | Yes |
| 865.5043 | 865.5025 | 2.080 | [M-H]- | PG 44:12 | PG | Yes | Yes |
| 887.5605 | 887.566 | -5.633 | [M-H]- | PI (18:0/20:3)* | PI | Yes | Yes |

*Confirmed by MS/MS experiment

**Supplementary Table 4.** Molecules identified in PanCK^-^CD105^-^ regions in tumoroids of case 561 and 622.

| *m/z* | Matched *m/z* | ppm error | Ion | Molecule | Class | **Case**  **561** | **Case 622** |
| --- | --- | --- | --- | --- | --- | --- | --- |
| 705.4925 | 705.4865 | 8.505 | [M-H]- | PA O-38:6 | PA | Yes | Not seen |
| 707.5013 | 707.5021 | -1.131 | [M-H]- | PA O-38:5 | PA | Yes | Yes |
| 748.5275 | 748.5287 | -1.603 | [M-H]- | PE O-38:6 | PE | Yes | Yes |
| 749.5304 | 749.5338 | -4.536 | [M-H]- | PG 34:0 | PG | Yes | Yes |
| 750.5426 | 750.5443 | -2.265 | [M-H]- | PE O-38:5 | PE | Yes | Yes |
| 752.5217 | 752.5236 | -2.525 | [M-H]- | PE 37:4 | PE | Yes | Not seen |
| 810.5301 | 810.5291 | 1.234 | [M-H]- | PS 38:4 | PS | Yes | Yes |
| 866.595 | 866.5917 | 3.808 | [M-H]- | PS 42:4 | PS | Yes | Not seen |
| 885.5495 | 885.5499 | -0.452 | [M-H]- | PI (18:0/20:4)* | PI | Yes | Yes |
| 886.553 | 886.5604 | -8.347 | [M-H]- | PS 44:8 | PS | Yes | Yes |
| 889.6189 | 889.6175 | 1.574 | [M-H]- | PI O-39:2 | PI | Yes | Yes |
| 891.6393 | 891.6332 | 6.841 | [M-H]- | PI O-39:1 | PI | Yes | Yes |

*Confirmed by MS/MS experiment

**Supplementary Table 5.** ROC analysis results – molecules capable of distinguishing regions of PanCK^-^CD105^-^Ki67^+^ and PanCK^-^CD105^-^Ki67^-^

| ***m/z*** | **Matched *m/z*** | **ppm error** | **Ion** | **Molecule** | **Class** | **AUC** | **Phenotype** |
| --- | --- | --- | --- | --- | --- | --- | --- |
| 700.5264 | 700.5287 | 3.283 | [M-H]- | PE O-34:2 | PE | 0.875 | PanCK-CD105-Ki67+ |
| 718.5344 | 718.5392 | 6.680 | [M-H]- | PE 34:0 | PE | 0.856 | PanCK-CD105-Ki67+ |
| 716.5233 | 716.5236 | 0.419 | [M-H]- | PE 34:1 | PE | 0.868 | PanCK-CD105-Ki67+ |
| 746.5172 | 746.513 | -5.626 | [M-H]- | PE O-38:7 | PE | 0.857 | PanCK-CD105-Ki67+ |
| 744.5538 | 744.5549 | 1.477 | [M-H]- | PE 36:1 | PE | 0.851 | PanCK-CD105-Ki67+ |
|  | 744.5493 | -6.044 | [M-2H]2- | CL 74:1 | CL |  |  |
| 890.6348 | 890.6397 | 5.502 | [M-H]- | SHexCer 42:1;O2 | SHexCer | 0.143 | PanCK-CD105-Ki67- |
| 890.6383 | - | - | - | - | - | 0.147 | PanCK-CD105-Ki67- |
| 862.608 | 862.6084 | 0.464 | [M-H]- | SHexCer 40:1;O2 | SHexCer | 0.118 | PanCK-CD105-Ki67- |
| 888.6253 | 888.624 | -1.463 | [M-H]- | SHexCer 42:2;O2 | SHexCer | 0.123 | PanCK-CD105-Ki67- |
|  | 888.6255 | 0.225 | [M+Cl]- | PE 44:3 | PE |  |  |
| 889.627 | - | - | - | - | - | 0.149 | PanCK-CD105-Ki67- |
| 860.5952 | 860.5942 | -1.162 | [M+Cl]- | PC 39:3 | PC | 0.135 | PanCK-CD105-Ki67- |
|  | 860.5942 | -1.162 | [M+Cl]- | PE 42:3 | PE |  |  |
| 897.5412 | 897.5499 | 9.693 | [M-H]- | PI 39:5 | PI | 0.135 | PanCK-CD105-Ki67- |
| 860.5759 | - | - | - | - | - | 0.142 | PanCK-CD105-Ki67- |

**Supplementary Table 6.** ROC analysis results – molecules capable of distinguishing regions of PanCK^+^CD105^+^Ki67^+^ and PanCK^-^CD105^-^Ki67^+^

| ***m/z*** | **Matched *m/z*** | **ppm error** | **Ion** | **Molecule** | **Class** | **AUC** | **Phenotype** |
| --- | --- | --- | --- | --- | --- | --- | --- |
| 777.5562 | 777.5571 | 1.157 | [M+Cl]- | PA O-40:2 | PA | 0.851 | PanCK+CD105+Ki67+ |
| 718.5344 | 718.5392 | 6.680 | [M-H]- | PE 34:0 | PE | 0.853 | PanCK+CD105+Ki67+ |
| 747.5169 | 747.5182 | 1.739 | [M-H]- | PG 34:1 | PG | 0.853 | PanCK+CD105+Ki67+ |
| 687.5414 | - | - | - | - | - | 0.858 | PanCK+CD105+Ki67+ |
| 835.5316 | 835.5342 | 3.112 | [M-H]- | PI 34:1 | PI | 0.858 | PanCK+CD105+Ki67+ |
| 717.5253 | 717.5258 | 0.697 | [M-2H]2- | CL 70:0 | CL | 0.862 | PanCK+CD105+Ki67+ |
| 716.5233 | 716.5236 | 0.419 | [M-H]- | PE 34:1 | PE | 0.869 | PanCK+CD105+Ki67+ |
| 746.5172 | 746.513 | -5.626 | [M-H]- | PE O-38:7 | PE | 0.872 | PanCK+CD105+Ki67+ |
| 776.5515 | 776.5447 | -8.757 | [M-H]- | PS 35:0 | PS | 0.875 | PanCK+CD105+Ki67+ |
|  | 776.5449 | -8.499 | [M+Cl]- | HexCer 36:2;O3 | HexCer |  |  |
| 746.5575 | - | - | - | - | - | 0.878 | PanCK+CD105+Ki67+ |
| 770.5681 | 770.5705 | 3.115 | [M-H]- | PE 38:2 | PE | 0.893 | PanCK+CD105+Ki67+ |
| 770.5613 | - | - | - | - | - | 0.894 | PanCK+CD105+Ki67+ |
| 745.556 | 745.5571 | 1.475 | [M-2H]2- | CL 74:0 | CL | 0.896 | PanCK+CD105+Ki67+ |
| 743.5414 | 743.5414 | 0.000 | [M-2H]2- | CL 74:2 | CL | 0.897 | PanCK+CD105+Ki67+ |
| 772.5319 | 772.5287 | 4.142 | [M-H]- | PE O-40:8 | PE | 0.902 | PanCK+CD105+Ki67+ |
|  | 742.5336 | -6.330 | [M-2H]2- | CL 74:3 | CL |  |  |
| 744.5538 | 744.5549 | 1.477 | [M-H]- | PE 36:1 | PE | 0.911 | PanCK+CD105+Ki67+ |
| 775.5455 | 775.5495 | 5.158 | [M-H]- | PG 36:1 | PG | 0.915 | PanCK+CD105+Ki67+ |
| 742.5383 | 742.5392 | 1.212 | [M-H]- | PE 36:2 | PE | 0.917 | PanCK+CD105+Ki67+ |
| 774.5389 | 774.5443 | 6.972 | [M-H]- | PE O-40:7 | PE | 0.924 | PanCK+CD105+Ki67+ |
| 843.5177 | 843.5182 | 0.593 | [M-H]- | PG 42:9 | PG | 0.027 | PanCK-CD105-Ki67+ |
| 845.5343 | 845.5338 | -0.591 | [M-H]- | PG 42:8 | PG | 0.032 | PanCK-CD105-Ki67+ |
| 871.5421 | 871.5473 | 5.966 | [M+Cl]- | PI O-35:1 | PI | 0.032 | PanCK-CD105-Ki67+ |
| 844.5217 | - | - | - | - | - | 0.036 | PanCK-CD105-Ki67+ |
| 841.5032 | 841.5025 | -0.832 | [M-H]- | PG 42:10 | PG | 0.047 | PanCK-CD105-Ki67+ |
| 869.5303 | 869.5338 | 4.025 | [M-H]- | PG 44:10 | PG | 0.048 | PanCK-CD105-Ki67+ |
|  | 869.5316 | 1.495 | [M+Cl]- | PI O-35:2 | PI |  |  |
| 846.5322 | 846.5291 | -3.662 | [M-H]- | PS 41:7 | PS | 0.051 | PanCK-CD105-Ki67+ |
| 842.5094 | 842.5108 | 1.662 | [M+Cl]- | PE 40:6;O | PE | 0.054 | PanCK-CD105-Ki67+ |
| 870.5345 | - | - | - | - | - | 0.059 | PanCK-CD105-Ki67+ |
| 844.5347 | - | - | - | - | - | 0.061 | PanCK-CD105-Ki67+ |
| 846.5396 | - | - | - | - | - | 0.064 | PanCK-CD105-Ki67+ |
| 872.5455 | - | - | - | - | - | 0.078 | PanCK-CD105-Ki67+ |
| 868.5201 | - | - | - | - | - | 0.102 | PanCK-CD105-Ki67+ |
| 867.5161 | 867.516 | -0.115 | [M+Cl]- | PI O-35:3 | PI | 0.105 | PanCK-CD105-Ki67+ |
| 762.5421 | - | - | - | - | - | 0.114 | PanCK-CD105-Ki67+ |
| 817.5054 | 817.5025 | -3.547 | [M-H]- | PG 40:8 | PG | 0.115 | PanCK-CD105-Ki67+ |
| 818.6267 | 818.6281 | 1.710 | [M-H]- | PS O-39:0 | PS | 0.115 | PanCK-CD105-Ki67+ |
| 762.5356 | 762.5291 | -8.524 | [M-H]- | PS 34:0 | PS | 0.120 | PanCK-CD105-Ki67+ |
|  | 762.5292 | -8.393 | [M+Cl]- | HexCer 35:2;O3 | HexCer |  |  |
| 763.5463 | 763.5495 | 4.191 | [M-H]- | PG 35:0 | PG | 0.125 | PanCK-CD105-Ki67+ |
| 847.5427 | 847.5495 | 8.023 | [M-H]- | PG 42:7 | PG | 0.131 | PanCK-CD105-Ki67+ |
| 818.5098 | 818.5108 | 1.222 | [M+Cl]- | PE 38:4;O | PE | 0.135 | PanCK-CD105-Ki67+ |
| 763.5377 | - | - | - | - | - | 0.136 | PanCK-CD105-Ki67+ |
| 890.6348 | 890.6397 | 5.502 | [M-H]- | SHexCer 42:1;O2 | Cer | 0.139 | PanCK-CD105-Ki67+ |
| 890.6383 | - | - | - | - | - | 0.140 | PanCK-CD105-Ki67+ |
| 810.5297 | 810.5291 | -0.740 | [M-H]- | PS 38:4 | PS | 0.145 | PanCK-CD105-Ki67+ |
| 819.6301 | - | - | - | - | - | 0.145 | PanCK-CD105-Ki67+ |
| 779.5441 | - | - | - | - | - | 0.147 | PanCK-CD105-Ki67+ |
| 891.6290 | 891.6332 | 4.710 | [M-H]- | PI O-39:1 | PI | 0.150 | PanCK-CD105-Ki67+ |

**Supplementary Method Information**

**Supplementary Table 7.** Summary of antibody dilutions

| ***Antibody*** | ***Supplier*** | ***Dilution*** |
| --- | --- | --- |
| *Hoechst33342* | *Fisher scientific* | *1:100* |
| *Ki67* | *Agilent* | *1:200* |
| *CD45* | *Abcam* | *1:500* |
| *Cleaved caspase 3* | *Cell Signaling* | *1:500* |
| *CD20* | *Agilent* | *1:50* |
| *CD3* | *Agilent* | *1:50* |
| *Vimentin* | *Cell Signaling* | *1:400* |
| *P53* | *Agilent* | *1:50* |
| *CD68* | *Abcam* | *1:3000* |
| *CD56* | *Cell Signaling* | *1:500* |
| *CD11b* | *Abcam* | *1:1000* |
| *ICOS* | *Abcam* | *1:500* |
| *MCK (AE1/AE3)* | *Agilent* | *1:100* |
| *Cytokeratin (Wide spectrum)* | *Agilent* | *1:100* |
| *CD105* | *Human Protein Atlas* | *1:600* |
